# ‘I am all alone’: factors influencing the provision of termination of pregnancy services in two South African provinces

**DOI:** 10.1080/16549716.2017.1347369

**Published:** 2017-08-08

**Authors:** Mantshi E. Teffo, Laetitia C. Rispel

**Affiliations:** ^a^ School of Public Health, Faculty of Health Sciences, University of the Witwatersrand, Johannesburg, South Africa

**Keywords:** Abortion, termination of pregnancy, health care provider, South Africa

## Abstract

**Background**: Globally, universal access to sexual and reproductive health care services has been re-emphasised. One-third of maternal deaths could be averted by improving access to safe abortion services. Anecdotal evidence suggests that the implementation of the Choice of Termination of Pregnancy Act has been suboptimal in South Africa.

**Objectives**: In two South African provinces, determine: the proportion of designated termination of pregnancy (TOP) facilities that provide these services; explore the factors that influence the provision of TOP services; and explore the work experiences of health care providers at designated TOP facilities.

**Methods**: During 2014 and 2015, we conducted a cross-sectional study at designated TOP facilities in Gauteng and North West provinces. A combination of methods was used, consisting of: site visits to, and observation of, each of the designated facilities using a checklist, and in-depth interviews with a sub-set of 30 TOP service providers, using a semi-structured interview schedule. The interview questions focused on the factors influencing TOP service provision, and the work experiences of TOP service providers. We used interpretative phenomenological analysis to analyse the data from the interviews.

**Results**: Overall, 77% (47/61) of designated facilities were providing TOP services, with 87.5% (28/32) in Gauteng Province, compared with 65.5% (19/29) in North West Province. Service provision was influenced by health system deficiencies, human resource challenges, lack of prioritisation and lack of management support. Study participants reported a heavy burden of care provision and expressed an overwhelming feeling of loneliness, courtesy stigma and lack of support from other nurses and doctors, which further influence TOP service provision.

**Conclusions**: South Africa has an enabling legal environment for the provision of TOP services. Supportive management, prioritisation of TOP services and employee wellness programmes to address the psychosocial issues experienced by providers are critical elements of an enabling health policy environment.

## Background

Globally, efforts to achieve gender equality and to reduce high levels of maternal mortality were given renewed impetus with Millennium Development Goals 3 (gender) and 5 (maternal mortality) [1]. The target for MDG 5 was to achieve a 75% reduction of the maternal mortality ratio between 1990 and 2015 [1]. Notwithstanding encouraging progress on the reduction of maternal mortality by the end of 2015, thousands of women continue to die from unsafe abortion, the majority in low- and middle-income countries [2]. The 2015 Sustainable Development Goals emphasise the importance of universal access to sexual and reproductive health care services in order to prevent avoidable maternal deaths [3]. One-third of maternal deaths could be averted by addressing unmet family planning needs, and improving access to safe abortion or termination of pregnancy (TOP) services [4,5]. It has been estimated that the proportion of all unsafe abortions has increased between 1995 and 2008 [6], while the World Health Organization (WHO) has estimated that worldwide, around 22 million unsafe abortions take place every year, thus contributing to the global burden of maternal mortality and morbidity [7]. An adequately skilled and motivated health workforce remains the most important strategy to ensure women’s access to sexual and reproductive health services and a reduction of avoidable maternal deaths [7].

In South Africa, the Choice of Termination of Pregnancy Act (CTOPA) was promulgated in 1996, and amended in 2008, in recognition of women’s reproductive rights, and to contribute to an improvement in women’s health and a reduction in maternal mortality from illegal abortions [8]. A 2000 evaluation on the implementation of the CTOPA found that within the first three years of the promulgation of the Act, more than 40,000 women had benefited from the improved access to TOP services [9]. However, maternal mortality in South Africa remains unacceptably high at an estimated 155 per 100,000 live births in 2013 [10]. Difficulties in access to safe TOP services add to HIV and obstetric causes of maternal mortality [11]. The factors that have influenced access to TOP services include: negative attitudes towards TOP services and the staff who provide TOP services; poor infrastructure; staff shortages and lack of support programmes for the few health care providers in existing TOP services [12,13].

There is a significant body of literature on abortion, which remains a hotly contested and debated issue. Research on abortion has focused on: the legal and ethical standards for protecting women’s human rights [14–16]; global and regional estimates of the incidence of unsafe abortion and associated mortality [4,6]; the relationship between induced abortion and adverse psychological outcomes among women undergoing the procedure [17]; conscientious objection and health care providers’ attitudes towards TOP services [16,18]; and strategies to reduce the incidence of unintended pregnancy and unsafe abortion [19]. Studies among health care providers have reported feelings of anxiety, professional ambivalence and conscientious objection, experiences of courtesy stigma and discrimination, violence or fear of violence, often resulting in health care providers not talking about their work or not advocating for the provision of TOP services, and fear of being labelled or rejected [20–22]. These negative emotions or experiences are exacerbated by health system deficiencies, insufficient or lack of peer support, lack of understanding of the consequences of stigma for abortion providers, potential risks of the procedure, and request for abortion from women with advanced pregnancies [14,23,24].

In South Africa, studies conducted since the implementation of CTOPA have reported on the progress made in providing TOP services, notably the increase in the number of designated facilities and staff capacity-building, as well as the ongoing challenges of negative provider attitudes [9,25,26]. However, there has been insufficient scholarly focus on the psychosocial issues faced by health care providers of abortion services [27].

In light of the importance of health care providers to improving women’s access to safe abortion services, the objectives of this paper are threefold: to determine the proportion of designated TOP facilities in the public sector that actually provide these services; explore the factors that influence the provision of TOP services; and explore the work experiences of TOP providers at designated facilities in two South African provinces. The research forms part of a larger doctoral study on psychosocial issues experienced and coping strategies used by health professionals that provide TOP services.

## Methods

### Study setting

The study was carried out at designated TOP facilities in Gauteng, an urban province, and North West, a mixed urban-rural province. These two provinces were selected because of geographical proximity to the researchers, logistical considerations and budgetary constraints.

### Study design

During 2014 and 2015, we conducted a cross-sectional study that used a combination of methods: site visits to and observation of all designated TOP facilities in Gauteng and North West provinces, using a checklist, surveys among TOP service providers and facility managers, and in-depth interviews with a sub-set of TOP service providers.

In this paper, we only focus on the site visits to and observation of all designated TOP facilities and the in-depth interviews with TOP service providers.

### Sampling, study population and recruitment

The sampling frame consisted of all public health facilities (hospitals and clinics) in Gauteng and North West provinces that are designated by the national Department of Health (DoH) to provide TOP services, whether as stand-alone or as integrated sexual and reproductive health services. We obtained a 2014 list of these designated TOP facilities from the national DoH, which was compared with the 2014 list from the provincial database from each of the study provinces. A designated facility is defined as one that meets the requirements to provide TOP services in terms of section 3 of the CTOPA, and is certified as such by the national DOH [28]. In each province, we verified the provincial database by interviewing the overall manager responsible for all sexual and reproductive health services.

The population of interest was all TOP health care providers, defined as professional nurses (with four years of training) or medical doctors (practitioners), whether full time or part time, providing TOP services in the public health sector at the designated facilities in the two provinces.

We selected a sub-set of 30 TOP service providers from the designated facilities that included a mix of Gauteng and North West hospitals and clinics, for in-depth interviews, using a purposive sampling technique.

### Ethical considerations

The University of Witwatersrand’s Human Research Ethics Committee (Medical) provided ethics approval for the study. The relevant provincial health authorities, including hospital and health centre managers, also provided study approval. We adhered to standard ethical requirements including participant-informed consent, detailed study information sheets, voluntary participation and maintaining confidentiality of information.

### Data collection

We designed a spreadsheet/facility checklist for the site visit and observation with the following details: province identification number, name of the district, facility number, health care provider identity number, whether TOP services were provided, and the number and category (professional nurse or doctor) of TOP providers at each facility.

The principal researcher visited each of the designated facilities between July 2014 and August 2015. In addition to the information on the spreadsheet, the principal researcher took detailed field notes. In those instances where TOP services were not provided at a designated facility, the researcher recorded the stated reasons for non-provision. The researcher also ascertained whether there were any facilities that were not on the original list of designated facilities, but that were providing TOP services. These additional facilities were also visited, and detailed field notes were recorded.

We designed a semi-structured interview schedule to explore the work experiences of TOP providers and their perspectives on the factors that influence TOP provision. The questions focused on: perceptions of their work as TOP providers, including rewarding and challenging aspects, psychosocial issues faced in the workplace, coping strategies, availability of support mechanisms in the workplace, the factors that influence TOP provision, and their recommendations for change.

The principal researcher invited the selected TOP providers to participate in the interview, and gave each person an information sheet and consent form. She explained the voluntary nature of the study, informed participants that no names would be used during the interview, and assured them of confidentiality and anonymity. Following informed consent, the researcher conducted semi-structured interviews with these TOP providers in English, which is the official business language of South Africa.

Each interview lasted around 40 minutes, although the length of time ranged from 30 minutes to 60 minutes. The researcher also took detailed notes during the interview and wrote a synopsis of each interview.

### Data analysis

The information from the site visits was collected on a Microsoft Excel spreadsheet, and this programme was used to analyse the data from the site visits.

We transcribed the recorded interviews verbatim. Data cleaning took one month and consisted of an iterative process of checking the transcribed interviews against the original recordings, correcting the text, checking the recordings again and making final corrections. Prior to analysis, an audit of provider interviews was done, and each provider was allocated a code number to ensure confidentiality of information. The coded interviews were consolidated into one file for ease of analysis.

We used interpretative phenomenological analysis for the analysis of the semi-structured interviews with TOP providers [29,30]. To ensure reliability, two other researchers (one a medical anthropologist and the other the research supervisor of the principal investigator) participated in the development of the themes by reading four diverse transcripts. Each researcher examined the meaning of the words of participants, and developed a set of codes, rather than using a pre-existing theory to identify codes that could be applied to the data [29,30]. We held a meeting of the three researchers to discuss the codes and to reach agreement on the codes and themes, namely the recurring patterns of meaning (ideas, thoughts and feelings) that emerged throughout the provider interviews. The principal investigator merged the codes and themes into one consolidated code book, prior to analysing the data using MAXQDA® 12.

## Results

### Provision of TOP services by designated facilities

In 2014, the number of designated TOP facilities in the two provinces was 61. Fourteen of the designated facilities did not provide TOP services, while we found four facilities in North West Province that were not on the national database, but providing TOP services. Hence the total number of facilities that were providing TOP services was 51 (Table 1).

**Table 1.** TOP service provision in Gauteng and North West provinces.

Indicator	Gauteng	North West	Total
Facilities designated to provide TOP services according to national database	32 (52%)	29 (48%)	61 (100%)
Facilities designated but NOT providing TOP services	4 (28.6%)	10 (71.4%)	14 (100%)
Facilities not on national database, but providing TOP services	0	4 (100%)	4 (100%)
Facilities providing TOP services	28 (55%)	23 (45%)	51 (100%)
Type of facility providing TOP services			
Hospital	16 (57%)	10 (43%)	26 (100%)
Community health centre	12 (43%)	13 (57%)	25 (100%)
Proportion of designated facilities that provide TOP services	28/32 (87.5%)	19/29 (65.5%)	47/61 (77%)

As can be seen from Table 1, 28/32 (87.5%) of designated facilities in Gauteng Province, and 19/29 (65.5%) of designated facilities in North West Province, provided TOP services. This means that 47/61 (77%) of the original designated facilities on the national database provided TOP services. If the four TOP facilities in North West are included, the proportion of designated facilities providing TOP services increases to 83.6%.

### Reported reasons for non-provision of TOP services

There were three reported reasons by the 14 facilities that did not provide TOP services in the two provinces: human resource challenges, health system deficiencies and lack of management support.

The human resource challenges included: TOP provider resignation or retirement, and not being replaced by other staff; and allocation of nurses to other health programmes, or sending them for training to support other programmes (such as primary health care), because TOP services were not considered to be a priority. Health system deficiencies included the lack of equipment such as sonar machines or manual vacuum extractors, and lack of space to provide services. The lack of management support for TOP services meant that managers were often reluctant to recruit staff or procure equipment or supplies for TOP service provision. During the fieldwork to the 51 facilities, the principal researcher personally observed the lack of management interest and support at the health care facilities. Some managers did not know when TOP services were provided, how many clients on average were seen per day, the type of psychosocial support TOP providers get if any, and the difference between first- and second-trimester protocols. In some instances the animosity between the manager and TOP provider was evident, and verbalised by providers or managers.

### Profile of TOP service providers interviewed

A total of 30 TOP providers were interviewed, 12 in Gauteng and 18 in North West Province. All the respondents were professional nurses, the majority were female (26/30 = 86.6%), with a mean age of 45.8 years. The TOP provision experience of participants ranged from 1 to 13 years.

### Work experiences and perspectives of health care providers on TOP service provision

Although inter-related, five major themes emerged from the interviews with TOP providers: rewarding aspects of the job, relationships with colleagues, unsupportive management, lack of an enabling environment and the emotional impact of TOP provision. The themes and sub-themes are shown in Table 2 and reported on separately for the sake of clarity.

**Table 2.** Themes emerging from the interviews.

Theme	Sub-theme
Rewarding aspects of the job	Preventing complications Preventing teenage or unwanted pregnancy Providing professional help to prevent complications and reduce client stress Understanding people’s problems Duty to provide TOP
Negative relationships with colleagues	Name-calling and stigmatisation Colleagues’ negative attitudes Unsupportive colleagues Being undermined by other colleagues Refusal to provide TOP services despite being trained Lack of medical doctor support
Unsupportive management	TOP services not prioritised No interest from management Lack of support No appreciation or rewards for work done No debriefing or support services provided
Lack of enabling environment	Lack of equipment, e.g. sonar machines Lack of medication Lack of space Large workload Staff shortages
Emotional burden of TOP provision	Loneliness of TOP provider Emotional trauma/traumatic experience Feeling demoralised

#### Rewarding aspects of the job

The majority of the providers considered it their professional duty and responsibility to provide TOP services. In response to the question on the rewarding aspects of their work, participants reported that they get satisfaction from their ability to understand and respond sensitively to women’s health care needs, prevent teenage or unwanted pregnancies, ensure access to safe services and prevent complications of backstreet abortions, such as septicaemia, hysterectomy and even death. The sense of meaning that TOP providers attach to their work is reflected in the two quotes, one from each of the study provinces, below:

'Seeing our young and black sisters dying from doing backstreet abortion, I decided to join TOP and help them to do medical TOP in a professional manner … That is how I developed the love for TOP; it was so I could help these young ones … Secondly some kids at schools have their future doomed by unwanted teenage pregnancies or staying at home with unwanted babies; they could have aborted the babies and continued with their studies and have babies later on after completing their education … or when they are being financially stable and mentally mature and ready.' (Respondent 11, North West Province)

'Helping children that come for TOP who are mostly rape survivors... Again helping parents make a choice for their young and immature children … One cannot help but want to spare the girl the pain and suffering especially when she refuses abortion saying she wants to keep the baby to play with her.' (Respondent 20, Gauteng Province)

#### Negative relationships with colleagues

The second major theme that emerged was the negative relationships with colleagues. The study participants reported judgemental attitudes from their colleagues, labelling, and being called names such as ‘murderers, killers, baby killers’. They reported that their colleagues often mocked them and asked how they (TOP providers) are able to sleep at night. The quotes below are illustrative of TOP provider narratives.

'We get labelled as killers as we get associated with all sorts of bad to say the least … The patient comes to seek service and they will just say to [name] those are your people, they [other colleagues] do not want anything to do with them [women requesting abortion]. We also get judged and people think that I am promoting promiscuity by doing TOP.' (Respondent 12, North West Province)

'For a long time I was called an undertaker, a killer amongst other things.' (Respondent 14, North West Province)

'The problem starts when we knock off at 4 pm and colleagues in the ward must take care of these patients … they get upset because they are not ‘killers’. They say ‘I am not going to touch them because I am not a killer’… (Respondent 27, Gauteng Province)

In some instances, participants reported that their colleagues discourage clients from obtaining TOP services and refuse to support TOP clients or to provide services even though they might have received TOP training. They reported that many of their colleagues were unsupportive, undermined their work and made sarcastic comments about them. Their colleagues also say that ‘TOP is a deterrent to family planning service provision’. This attitude and lack of understanding of TOP processes often resulted in conflict and strained relationships between TOP providers and their other nursing colleagues, and one indicated that she gets no support ‘from my colleagues, especially not in this ward, oh here I am in hell’ (respondent 27, Gauteng Province).

Others said the following:

'Ahhhaaa … there is no appreciation from colleagues at all, you will never get assistance and that is why I am always there alone, there is totally no appreciation from colleagues.' (Respondent 13, North West Province)

'When you’re alone and having to turn away patients; that is just not right because the patient has nothing to do with staff shortages and it is so unfair for the patients.' (Respondent 20, Gauteng Province)

The TOP service providers reported that medical support for TOP services was inadequate, and that there were few doctors available to assist with emergencies. Some doctors, despite being trained, refused to be called TOP providers, and sometimes shifted their responsibility of managing complications and second-trimester TOPs to the nurse providers. At some facilities doctors reportedly send patients from pillar to post, and even refer patients to a referral facility that does not provide TOP. In North West Province, one doctor refused to do a sonar, saying they (women requesting abortion) should have prevented the pregnancies if they did not want babies. The study participants indicated that they often had to ‘fight’ before they could get a facility manager or doctor to respond to requests for assistance.

#### Unsupportive management

The third major theme that emerged from the interviews was unsupportive facility management. Participants reported that hospital or community health centre managers showed little appreciation for the work that they were doing, or an interest in knowing about TOP. Providers reported that they were often left all alone ‘in their little corner’ and nobody cared about them. Providers were frustrated that managers were supposed to support them, but instead these managers did not visit the TOP units, they did not address interpersonal relationship issues affecting TOP service provision and did not take time to find out whether the few TOP providers were coping with their jobs.

While some providers attended one debriefing session at the end of the year, some reported that they never had any debriefing sessions in the preceding years. The study participants reported that the facility managers only visited the unit when they (managers) wanted statistics, followed up on a complaint or showed visitors around. One of the TOP providers reported that a Gauteng facility manager was overheard saying that ‘if TOP units were shut down, nobody would die, and other patients would use that space’.

Participants reported that they are often called upon to relieve in other wards or units, because TOP services are not prioritised. The unsupportive management and lack of prioritisation of TOP services in turn increase the stress and workload of existing TOP providers.

'My major issue is that as an advanced midwife, I work at all units in the facility especially labour ward and relieve the operational manager … Additionally I am the only one trained to insert and remove IUCD and implants. My hands are full … In case of an emergency I am expected to leave the TOP unit and run. I become confused as I am expected to be everywhere … TOP is not prioritised anyway.' (Respondent 29, North West Province)

'I am all alone, it is just me. It is difficult because I play a dual role as both the provider and the manager … I work within a structure that is not well equipped … my colleagues are judgemental and look down on me as a TOP provider … Sometimes you come across situations where you really should consider leaving the client but then again if you leave her you realise that the person is going through a lot, you cannot deny her the service. There is no social worker or psychologist at the hospital to support clients.' (Respondent 5, North West Province)

'The Department [of Health] needs to realise that TOP is one of their services … We didn’t initiate this service, it was initiated by the department because there was a need but now it is like it addresses our need and we have to see to finish... Patients come for a service whether or not there is a provider.' (Respondent 15, North West Province)

#### Lack of an enabling environment

Participants reported on the lack of an enabling environment that ranged from the unavailability of medication, malfunctioning or unavailable equipment especially sonar machines, lack of working space and staff shortages, all of which increase workload. The lack of medication in turn affects the women who request TOP services, as they have to return to the facility, as shown by the quote below:

'The problem is when we don’t have medication because there are those times when Misoprostol is finished and then people will have to go back because we don’t have medication.' (Respondent 6, North West Province)

TOP providers also complained about equipment, and one said:

'honestly we lack equipment and even the beds malfunction when it comes to adjusting them up and down. Some of these beds are very old.' (Respondent 22, Gauteng Province)

TOP providers reported that they were expected to share work space and/or sonar machines with other units, and in some instances they had to take clients to other units to do sonars. The lack of equipment impacts on client privacy and confidentiality, as illustrated by the quote below:

'Lack of equipment and supplies and having to move with patient to maternity for a sonar. When we get there everybody already knows what they have come for. There is no privacy at all … I also have to carry instruments from theatre to wash at the TOP unit.' (Respondent 28, Gauteng Province)

The lack of equipment also resulted in more time spent in preparations rather than the actual service provision, and resentful colleagues who felt that their work was delayed by sharing the sonar machine.

Respondents complained about staff shortages and about working alone. In some instances, women had to wait for abortions until the TOP providers returned from sick leave, which was stressful, and respondents indicated that this increased provider workload. Inadequate management support and delayed or non-supply of essential equipment were adding to the perceptions of a disabling environment.

'There is just too much that goes in TOP service … we are the only two providers, when the patient comes through we have to conduct sonar to examine the gestation … only when the patient qualifies do we then counsel, do observations only then book the patient … On the day of admission, you do the same procedure and initiate TOP with pills … you have to prepare or do the procedure and provide post abortion care … while you attend to other issues in between. Phew!' (Respondent 19, Gauteng Province)

#### Emotional burden of TOP provision

Notwithstanding the rewarding aspects of TOP service provision, and the desire to make a difference, participants reported that the strained relationship with colleagues, unsupportive management and lack of an enabling environment take an emotional toll on them. An overwhelming negative emotion expressed was one of ‘loneliness’.

'unfortunately people are not interested in knowing what we do … we are just left all alone in our little corner and nobody cares about us and what we do.' (Respondent 12, North West Province)

Participants reported that the constant reminders by colleagues and comments that ‘TOP is a dirty job and providers are killers’ were traumatic. At times the participants reported that they experience contradictory feelings of lack of interest or regret that they are TOP providers.

'More people must be trained because this is stressful … we volunteer to come to this department but after some time you feel as if you have made a mistake, you think you need a break but then again no one will come … [hence] you are forced to stay in the TOP department.' (Respondent 18, Gauteng Province)

'I am no longer interested to work in this department. Truly speaking it is stressful and there is no appreciation or no incentive like OSD [occupation specific dispensation, a public health sector financial incentive] or additional money.' (Respondent 25, Gauteng Province)

The study participants reported examples of TOP providers who have left because they could not handle the stigma and discrimination from other colleagues and managers. In addition, some study participants said that the lack of understanding from colleagues, the lack of management support and the emotional experience result in ‘abnormal coping mechanisms’ such as staying away from work without authority, request to be transferred to other departments, dreading to go to work on some days or not wanting to listen to anybody’s story.

'Normally I stay away from work, I request some days off but if they don’t give me those days off … I will just stay by myself just to relax … because I have asked them at least once in a month to go for debriefing sessions whether either a psychologist or someone … but they don’t allow that I have been going there by myself.' (Respondent 16, North West Province)

The providers reported that they were disturbed by manual vacuum aspiration, which is quite mechanical, the associated pain women experience during the procedure, the dangers of doing the procedure with a possibility of uterine rupture or subsequent death, and the fact that the procedure makes them see the limbs, hands and arms of dead foetuses, which they find very traumatic. They mentioned that the loss of sleep because of the traumatic experience, and the inability to share their feelings with colleagues made it more difficult. At the same time, the majority of providers indicated that they keep their emotions to themselves for fear of judgement or prejudice.

'I have to introduce the cannula right into the cervix, that’s when the woman starts to feel more pain when I am doing the aspiration and I don’t know if I am damaging her inside and when they are in pain that’s when it affects me the most.' (Respondent 16, North West Province)

'Sometimes we get burnout … you feel like resigning … or if not resigning you feel like going on leave for a very long time. If I can make an example, last week I was talking to my colleague telling her that I had a bad dream … This week I dreamt of doing the procedure which is not nice, we sleep knowing that we are doing TOP but we don’t want to wake up to it you know.' (Respondent 18, Gauteng Province)

## Discussion

This empirical study was conducted to explore TOP service provision by designated facilities, the factors that influence the provision of such services, and the work experiences and perceptions of TOP providers in two South African provinces.

This study found that 77% of designated facilities (or 84% when four from North West were included) in the two provinces were providing TOP. At face value, it is encouraging that the majority of designated facilities in the two provinces actually provided TOP services, as intended. More designated facilities were providing TOP services in the urban province of Gauteng (87.5%), compared with the mixed urban-rural province of North West (65.5%). The higher proportion of functional TOP facilities in Gauteng is supported by information from the District Health Information System. In 2014, 18,465 TOPs were done in Gauteng Province, compared with 8294 in North West Province [31]. The differences could be due to the ease of geographical access and better financial and human resource availability in urban Gauteng [32].

We found that 14 designated facilities in the two provinces or almost one-quarter (23%) of the total number of designated TOP facilities (14/61) were not providing TOP services at the time of the study, with the majority of these in North West Province. A 2015 rapid review found that fewer than half of government-designated facilities provide abortion services, because of conscientious objection by health care providers [26]. However, our study, which used a combination of site visits, observation and in-depth interviews with health care providers, found that a complex set of overlapping factors influenced the provision of TOP services in Gauteng and North West provinces. In essence these factors were: lack of an enabling environment because of the many health system deficiencies, and human resource challenges, exacerbated by the lack of management support for TOP services.

Although the CTOPA in South Africa provides an enabling legal framework for safe abortion services, study participants complained about a range of health system problems, including the lack of equipment, lack of medication and lack of space. WHO has pointed out that access to safe abortion services is dependent on the availability of services, the manner in which they are delivered, well-equipped facilities and trained health care providers [33]. Other studies have also found that health system deficiencies hinder safe abortion services. A 2015 systematic review to analyse factors that influence first-trimester abortion services for women in high-income countries found that, despite supportive legislation, insufficient hospital resources, particularly in rural areas, were a major barrier to care and contributed to inequities in quality of and access to TOP services [23]. Studies in low- and middle-income countries found inadequate health care and infrastructure, and unavailability of medical abortion including restricted laws and high cost were barriers to accessing TOP services despite the feasibility, effectiveness and acceptability of medical abortion in low-resource settings [34,35].

An enabling environment which includes infrastructure, availability of medicines and equipment, has quality of care and lower cost benefits [36,37] and is essential for safe abortion care. South Africa’s progressive abortion legislation necessitates that provincial health authorities ensure that systems are in place for procurement and distribution of all medical equipment, drugs and supplies necessary for the safe delivery of TOP services.

WHO has pointed out that the lack of trained health care providers is one of the most critical barriers to safe abortion care [7]. A 2015 systematic review on factors influencing the provision of TOP services in high income countries that found that lack of trained providers was an obstacle to safe abortion care [23]. In our study, professional nurses were the primary TOP providers. It has been pointed out that the implementation of the CTOPA in South Africa would not have been possible without the extensive support and involvement of a small group of committed nurses [27]. Our study participants complained of excessive workload and staff shortages. Other South African studies have also found a severe shortage of willing TOP providers [12,38]. These shortages were exacerbated by lack of management support observed and reported in the majority of designated facilities. The provincial health authorities, together with health facility managers, have the responsibility of ensuring resource availability, staff support and supervision, and procurement of needed equipment and medication [10]. They are also responsible for protecting the rights of women seeking TOP services and ensuring quality of care is provided. They must oversee and enforce accountability by doctors and nurses towards their professional and ethical responsibilities for TOP provision, with consequences if their duties are neglected.

An overwhelming feeling expressed by TOP providers was one of loneliness and lack of support from their nursing and medical colleagues. These TOP providers reported the heavy burden of care provision, which they felt even when off-duty. They lamented the unrealistic expectations from facility managers regarding assisting with nursing duties in other units. Their experiences of stigma and discrimination added to the health system barriers to TOP service provision. All these factors combined to take a huge emotional toll on TOP service providers, despite their accounts of the rewarding aspects of the job. They reported that their willingness to become TOP providers and commitment to safe abortion care were met with judgemental attitudes from their colleagues, labelling and name-calling such as murderers, killers, baby killers. These provider experiences of courtesy stigma, or stigma by association, increased their feelings of loneliness, and often resulted in conflict and strained relationships between TOP providers and other nurses. The refusal of other colleagues to provide TOP or to refer women to access TOP services added to provider loneliness. Furthermore, the non-responsiveness of doctors in providing clinical or referral support, and from managers for equipment, additional staff or medication, left many of the few TOP providers despondent.

There is evidence of loneliness experienced by women who choose TOP [17,39]. Many authors have reported on the problems of conscientious objection and the unwillingness of doctors or midwives to provide TOP services [16,18]. Similarly in South Africa, scholars have highlighted the access barriers experienced by women seeking safe abortions [18,26,40]. However, there is a dearth of studies in South Africa on the *loneliness* or the psychosocial issues experienced by providers of TOP services.

In this study, participants gave a moving account of their experiences of courtesy stigma from their colleagues and health facility managers. The sociologist Goffman coined the term ‘courtesy *stigma*’ and defined it as the stigma or public disapproval of those people who are merely associated with a *stigmatised* disease, person or group [41]. In the case of abortion, courtesy stigma is the name-calling or discrediting of individuals (in this case, health care providers) as a result of their association with women who request or are in need of abortion or TOP services [42]. In Ghana, a 2016 study also found that courtesy stigma resulted in health care provider reluctance to provide TOP [22]. In the USA, Harris et al. advanced the concept of courtesy stigma by describing both the negative stereotypes of abortion providers and the multiple levels at which providers experience stigma, as well as developing tools to measure abortion stigma [43]. Importantly, abortion provider stigmatisation has implications for the psychosocial well-being of TOP providers, availability of scarce human resources, patient safety and health policy [21].

The solutions to the problems reported by study participants require decisive actions from South African policy-makers, health service managers and gender activists. Similarly to efforts to combat HIV stigma and discrimination [44], strategies should include social mobilisation programmes and advocacy for women’s rights in the health sector and in society at large.

There are limitations to this study, which was undertaken among a sub-sample of 30 TOP service providers in two South African provinces. The cross-sectional nature of the study means that we obtained the views and perceptions of the providers at a point in time.

Nonetheless, this study is novel in conducting multi-site visits to 61 TOP facilities to determine whether designation means actual provision. The triangulation of data from the site visits, observation, combined with the narratives and perspectives of TOP service providers, give unique insights into TOP service provision. This gives an opportunity to revisit and address TOP service provider needs, and to ensure that the enabling provisions of CTOPA in South Africa are implemented. Another matter requiring attention is the re-introduction of value clarification workshops and training of healthcare workers on CTOPA, women’s rights, and the ethical duties and responsibilities of health professionals. Lastly, the study findings suggest the need for employee wellness programmes that take into account the psychosocial needs of TOP providers.

## Conclusions

South Africa has an enabling legal environment for the provision of TOP services. The study finding that three-quarters of designated facilities were functional as TOP facilities is encouraging. At the same time, this study highlighted the multiple, and overlapping, barriers to TOP service provision that included health system deficiencies, human resource challenges, lack of prioritisation and insufficient management support. The experiences of courtesy stigma and feelings of loneliness further threaten safe abortion services provided by a few willing and committed health workers. Although strengthening health systems and human resources is important, supportive management and prioritisation of TOP services are critical elements of an enabling health policy environment.

## Paper context

Previous research on this topic has focused on access barriers to the provision of termination of pregnancy (TOP) services, and conscientious objection by health care providers. This paper adds new knowledge on the loneliness experienced by TOP health care providers, and their experiences of courtesy stigma from their colleagues and managers. We recommend prioritisation of TOP service provision, employee wellness programmes and supportive management to address the psychosocial issues experienced by these providers.
